# Supplementary material for: The use of artificial intelligence in induced pluripotent stem cell-based technology over 10-year period: A systematic scoping review
Source: PLoS One. 2024 May 21;19(5):e0302537. doi: 10.1371/journal.pone.0302537 (PMC11108174; doi:10.1371/journal.pone.0302537)
Supplement: S1 File — (DOCX) [file pone.0302537.s002.docx]

**The use of Artificial Intelligence in induced pluripotent stem cell-based technology over 10-year period: A Systematic Scoping Review**

**Search query**: "Artificial Intelligence" OR "Machine Learning" OR "Deep Learning" OR "AI algorithms") AND ("induced Pluripotent Stem Cells" OR "iPSC" OR "iPSCs")

**S1 Table**. General information of included studies

| **No** | **First author** | **Year** | **Region** | **Cell** | **Cell type** | **Cell organ** | **Disease Model** | **Reseach classification** | **Input** | **Study analysis** | **Algorithm** | **Finding** | **Accurracy** |
| --- | --- | --- | --- | --- | --- | --- | --- | --- | --- | --- | --- | --- | --- |
| 1 | Aixia Sun [1] | 2021 | USA | hiPSC | organoid | islet | Type 1 diabetes | Cell processing | image | Cell tracking | K-means++ | ML can monitor the transplanted islet organoids longitudinally with indications on total iron content within subjects in a 3D manner | ICC score of 0.898 and 0.927 for single and average measures |
| 2 | Anna Skorska [2] | 2022 | Europe | iPSCs | cell | cardiomyocytes |  | Cell processing | image | Cell biological evaluation | CNN | AI-based imaging workflow can be used as a straight-forward tool to quantitatively estimate the structural maturation of contractile cell | Not report |
| 3 | Aurore Vuidel [3] | 2022 | Europe | hiPSC | cell | neuron | Parkinson | Cell processing | image | Cell classification | SVM, linear discriminant analysis | ML can be used to distinctively classify Parkinson disease from control | Accuracy was 0.98 ± SEM 0.02 |
| 4 | Bianca Williams [4] | 2020 | USA | hiPSC | cell | cardiomyocytes |  | Cell processing | Biological information | Cell classification | random forest, Gaussian process regression, multivariate adaptive regression spline | ML techniques can be employed using existing data for understanding and improving production of a specific cell type, which is potentially applicable to other lineages and critical for realization of their therapeutic applications. | Accuracy of 89%, precision and recall near 90%, and MCC values of 0.72. |
| 5 | Bo Jiang [5] | 2015 | China | hiPSC | cell | neural progenitors |  | Cell processing | image | Cell classification | SVM | ML has a remarkable recognition and generalization performance in classifying NPC under bright-field microscopic imaging | With the percentage of training samples decreasing, the accuracy of test is reduced from 100% to 56.8% |
| 6 | Brodie Fischbacher [6] | 2020 | USA | hiPSC | cell |  |  | Cell processing | image | Cell classification | CNN | DL algorithms with a modular design can automate the verification of monoclonality in bright-field microscopy, requiring relatively little labelling | Detection rate was 100% for colonies of sufficient size for passaging suggests |
| 7 | Cesar A. Patino [7] | 2020 | USA | iPSCs | cell |  |  | Cell processing | image | Cell evaluation | CNN | Automated single-cell delivery is a useful cell manipulation tool for applications that demand throughput, control, and precision | The segmentation performance of the automatically generated labels approached the precision of human annotations [F1auto (70% IOU) = .94] |
| 8 | Cesar A. Patino [8] | 2022 | USA | hiPSC | cell |  |  | Cell processing | image | Cell classification | CNN | The DL-based image analysis enabled the correlation of experimental inputs to the phenotypic outputs across devices in the multiwell LEPD | Not report |
| 9 | Chia-Chen Hsu [9] | 2020 | UK | hiPSC | cell | neuron |  | Cell processing | image | Cell classification | SVM, random forest, k-nearest neighbor | ML has high sensitivity, high specificity, and high accuracy of cell classification during different stages of neural development | The overall accuracy rate is as high as 97.5%. |
| 10 | Chung-Yueh Lien [10] | 2023 | Taiwan | hiPSC | cell | retinal |  | Cell processing | image | Cell classification | CNN | ML achieved a high accuracy in distinguishing between the iPSCs and iPSC-derived MSCs, RGCs, and RPEs | The overall accuracy rate is over 97% |
| 11 | Colombine Verzat [11] | 2021 | Europe | hiPSC | cell | neuron |  | Cell processing | image | Cell classification | CNN | DL methods for rapid, automated and unbiased identification of biological hypotheses | Not report |
| 12 | Dai Kusumoto [12] | 2018 | Japan | hiPSC | cell | Endothelial cells |  | Cell processing | image | Cell classification | CNN | DL techniques are effective in identifying iPSC-derived endothelial cells | The overall accuracy rate is over 80% |
| 13 | David A. Joy [13] | 2021 | USA | hiPSC | cell |  |  | Cell processing | image | Cell biological evaluation | CNN | Computational celltracking pipeline allows for a more comprehensive dynamic understanding of the multicellular principles of morphogenesis, which can empower more refined control of organoid and engineered tissue development. | AUC: 0.8 |
| 14 | Diogo Teles [14] | 2021 | USA | hiPSC | cell | cardiomyocytes |  | Cell processing | image | Cell classification | k-nearest neighbor, decision trees, t-SNE, Naïve Bayes classifiers | ML for the classification of diseased human iPS cells-derived cardiomyocytes. | Accuracies above 90% |
| 15 | Jianying Guo [15] | 2021 | USA | hiPSC | embroid |  |  | Cell processing | image | Cell biological evaluation | PhenoLOGIC machine learning | ML-assisted high-content 3D image analysis could accelerate the exploration of the stem cell-based embryo models as well as other organoid systems | Accuracy of correct classification reached 0.81, and the F1 score was 0.69 |
| 16 | Haishan Zhang [16] | 2019 | China | iPSCs | cell | cardiac progenitor |  | Cell processing | image | Cell classification | Boosting algorithm | ML can identify morphology and motion pattern of iPS progenitor cells | Accuracies above 60% |
| 17 | Henry Joutsijoki [17] | 2014 | Europe | iPSCs | cell |  |  | Cell processing | image | Cell biological evaluation | SVM, k-nearest neighbour, naïve Bayes, classification tree | The obtained results are promising for further research where, for instance, more sophisticated feature selection and extraction methods and other multi-class extensions of SVM will be examined. However, intensity histograms are not alone adequate for iPSC colony image classification | Accuracy ranged from 30-50% |
| 18 | Henry Joutsijoki [18] | 2016 | Europe | iPSCs | cell |  |  | Cell processing | image | Cell classification | SVM, k-nearest neighbor | ML can be used to identify when the patient’s somatic cells have been fully reprogrammed to iPSC and identify the quality of growing iPSC colonies in order to exclude the possible abnormal iPSC colonies | Accuracy ranged from 40-70% |
| 19 | Kaivalya Molugu [19] | 2022 | USA | hiPSC | cell |  |  | Cell processing | image | Cell classification | random forest, k-nearest neighbor, naïve Bayes, simple logistic | ML can predict the reprogramming status of cells, which could enable real-time monitoring during iPSC manufacturing, thereby aiding in the identification of high-quality iPSCs in a timely and cost-effective manner | Accuracy of 95% and model performance of 0.99 (AUC of ROC) |
| 20 | Kazuaki Tokunaga [20] | 2014 | USA | hiPSC | cell |  |  | Cell processing | image | Cell classification | weighted neighbour distances | ML was effective to classify partially reprogrammed and fully reprogrammed mouse cells grown in the same dish as a mixed population | Accuracy > 80% |
| 21 | Ke Fan [21] | 2017 | China | hiPSC | cell |  |  | Cell processing | image | Cell biological evaluation | CNN | ML algorithm-detected colonies show no significant differences (Pearson Coefficient) in terms of their biological features compared to the manually processed colonies using standard molecular approaches. | Accuracy > 99% |
| 22 | Ke Ye [22] | 2020 | Japan | hiPSC | cell | retinal |  | Cell processing | image | Cell quality control | hierarchical clustering | ML effective at identifying low-quality sheets for elimination, even when using label-free images | Accuracy: 99%, sensitivity: 98%, specificity: 100% |
| 23 | Ken Orita [23] | 2020 | Japan | hiPSC | cell | cardiomyocytes |  | Cell processing | image | Cell quality control | SVM | ML can precisely discriminate between functionally normal and abnormal contractions of hiPSC-CM. | Accuracy, precision, recall, and F1 score were 0.89 ± 0.02, 0.93 ± 0.04, 0.73 ± 0.05, and 0.82 ± 0.04, |
| 24 | Ken Orita [24] | 2019 | Japan | hiPSC | cell | cardiomyocytes |  | Cell processing | image | Cell quality control | CNN | CNN can assess the qualities of cultured hiPSC-CM | Accuracy, precision, recall, and F1 score were 0.897 ± 0.01, 0.946 ± 0.005, 0.843 ± 0.02, and 0.890 ± 0.01 |
| 25 | Keonhyeok Park [25] | 2022 | Korea | hiPSC | organoid | kidney |  | Cell processing | image | Cell classification | CNN | AI algorithm can successfully recognize the differentiation status of kidney organoids | Accuracy of 76.67% |
| 26 | Kimerly A. Powell [26] | 2023 | USA | hiPSC | cell |  |  | Cell processing | image | Cell quality control | U-Net segmentation model | DL provides a basis for investigating hiPSC morphology that is observed in early phase reprogramming images and correlating these features to the quality of the final downstream cell products. | DICE ranged from 0.68–0.96 |
| 27 | Letao Yang [27] | 2022 | USA | hiPSC | cell | hippocampus |  | Cell processing | image | Cell biological evaluation | Gaussian process regression | ML screening platform holds excellent potential in a wide range of biomedical applications from disease modeling to stem cell therapies and may thus enhance the treatment of various types of neurological disorders | Not report |
| 28 | Mahnaz Maddah [28] | 2018 | USA | hiPSC | cell | cardiomyocytes |  | Cell processing | image | Cell classification | SVM | ML imaging-based beating assay for characterization of stem cell-derived cardiomyocytes. | Not report |
| 29 | Minjae Kim [29] | 2023 | Korea | miPSC | cell |  |  | Cell processing | image | Cell biological evaluation | CNN | CNN can distinguish the subtle changes in the cell morphologies that could be occurred between the presence or absence of the small molecules that prevent abnormal gene expression patterns by looking only at cell images without the need for labeling. | Average accuracy of more than 90% |
| 30 | Muthu Subash Kavitha [30] | 2017 | Korea | iPSC | cell |  |  | Cell processing | image | Cell classification | SVM, random forest, decision tree, adaptive boosting classifier, Multilayer perceptron classifier | Automated fused statistical, shape-based, and moment-based texture pattern features trained with machine learning techniques are potentially more appropriate and helpful to biologists for characterizing colonies of stem cells | Highest predictive capability with maximum accuracy (91.6%), precision (92.0%), and recall (89.0%) |
| 31 | Na Ta [31] | 2020 | China | iPSC | cell |  |  | Cell processing | Genetic data | Cell tracking | CNN, SVM | AI can correctly identification of stage-specific gene clusters | The ROC ranged from 0.75 - 0.87 |
| 32 | Prithvijit Mukherjee [32] | 2022 | USA | hiPSC | cell |  |  | Cell processing | Genetic data | Cell biological evaluation | CNN | AI can provide superior cell viability and efficiency compared to traditional methods | Precision and recall of 91.4% and 97.9% |
| 33 | Quinton Smith [33] | 2018 | USA | hiPSC | cell |  |  | Cell processing | image | Cell classification | SVM | the applicability of AI technology for studying pattern formation in early and late-stage differentiation | Accuracy over 70% |
| 34 | Sally Esmail [34] | 2019 | Canada | aiPSC | cell | skeletal muscle cell |  | Cell processing | Genetic data | artificial iPSC | CNN, cognitive maps | DL potentially transform new technology to empower rare disease researcher | Precision = 94.59%, recall = 97.22%, F-Measure = 0.959 and Area under ROC curve (AUC) = 0.973 |
| 35 | Sally Esmail [35] | 2020 | Canada | aiPSC | cell | lung |  | Cell processing | Genetic data | artificial iPSC | CNN, cognitive maps | DL will improve preparedness for and response to future viral outbreaks. | mean of three independent experiments ± 99% confidence interval |
| 36 | Saori Aida [36] | 2019 | Japan | iPSC | cell |  |  | Cell processing | image | Cell biological evaluation | CNN | stablish a new drug discovery process. |  |
| 37 | Scott Atwell [37] | 2023 | Europe | hiPSC | cell |  |  | Cell processing | image | Cell classification | CNN | DL enables dynamic large-scale screening of 3D stem cell cultures based on bright-field imaging | Detection rate was 63.0% and detection precision was 44.0% |
| 38 | Slo-Li Chu [38] | 2022 | Taiwan | hiPSC | cell |  |  | Cell processing | image | Cell classification | CNN | DL efficiently select the reprogramming cells possibly forming hiPSCs and to predict the shapes of growing hiPSC colonies. | Accuracy over 80% and 66% of the identified cells under different culture conditions, |
| 39 | Takashi Waku [39] | 2017 | Japan | hiPSC | cell |  |  | Cell processing | image | Cell classification | SVM | AI aloe accurate classification, equivalent to visual inspection by experts | Accuracy over 80% |
| 40 | Tobias Piotrowski [40] | 2020 | Europe | hiPSC | cell |  |  | Cell processing | image | Cell classification | stepbased Stochastic Gradient Descent | AI can be exploited for routine, non-invasive image analysis during an automated hiPSC cultivation. | F1 score was 0.753 |
| 41 | Toshiro Iwagawa [41] | 2022 | Japan | hiPSC | cell | retinal |  | Cell processing | image | Cell biological evaluation | CNN | DL can be used to predict tissue function and the cellular donor identities of iPSC derived RPE | Accuracy over 90% |
| 42 | Yohei Hayashi [42] | 2018 | Japan | hiPSC | cell |  |  | Cell processing | image | Cell quality control | CNN | DL can be used to develop a fully automated culture system for human PSCs to ensure the quality and homogeneity of the cells | Not report |
| 43 | Yuan-Hsiang Chang [43] | 2017 | Taiwan | hiPSC | cell |  |  | Cell processing | image | Cell classification | CNN | DL can be used to help in continuous observations and automatic analysis of stem cells during reprogramming | Top-1 and Top-2 error rates of 9.2% and 0.84% |
| 44 | Wayne R. Danter [44] | 2019 | Canada | aiPSC | cell |  |  | Cell processing | Genetic data | artificial iPSC | CNN, cognitive maps | aiPSCs have the potential to improve disease modeling, prototyping of experiments, and prediction of genes relevant a | Accuracy over 90% |
| 45 | Alberto Catanese [45] | 2023 | Europe | hiPSC | cell | neuron | Amyotrophic lateral sclerosis | Genetic analysis | Genetic data | DNA sequences | CCN | AI provides significantly higher coverage of the transcriptional commonalities characterizing the different ALS cases | Not report |
| 46 | Asato Sekiya [46] | 2022 | Japan | hiPSC | cell | neuron |  | Genetic analysis | Genetic data | RNA sequences | maximum relevance minimum redundancy | ML can consider input–output nonlinear relationships and it is effective for high-dimensional data. | Not report |
| 47 | Boqiao Lai [47] | 2022 | USA | hiPSC | cell | neuron |  | Genetic analysis | Genetic data | Epigenomic assay | CNN | DL is a powerful framework of predicting epigenomic profiles from DNA sequences | AUROC of 0.87 and AUPRC of 0.82 |
| 48 | C Bardy [48] | 2016 | USA | hiPSC | cell | neuron |  | Genetic analysis | Genetic data | RNA sequences | randomized forest | MA transcriptome analysis can be used to predict the physiology of a cell | Good validation performance with 83% accuracy, ROC=0.93 |
| 49 | Christina V. Theodoris [49] | 2021 | USA | hiPSC | cell |  |  | Genetic analysis | Genetic data | RNA sequences | k-nearest neighbor | ML enabled the cost-effective interrogation | The overall accuracy rate is over 99% |
| 50 | Koichiro Nishino [50] | 2020 | Japan | iPSCs | cell |  |  | Genetic analysis | Genetic data | DNA sequences | SVM | ML model can distinguish among ESCs, iPSCs, embryonal carcinoma cells (ECCs), and somatic cells on the basis of their DNA methylation profiles | Precision, Recall, and F-score rates were 94.23%, 95.17%, 93.63% and 94.39% |
| 51 | Quan H. Nguyen [51] | 2018 | USA | hiPSC | cell |  |  | Genetic analysis | Genetic data | RNA sequences | clustering | AI can accurately classify single cells into each of the subpopulations | AI increases prediction accuracy by 10%, specificity by 20% |
| 52 | S John Liu [52] | 2017 | USA | hiPSC | cell |  |  | Genetic analysis | Genetic data | RNA sequences | SVM, random forest | ML can conduct large-scale screens for lncRNA function in multiple cell lines |  |
| 53 | Thong Ba Nguyen [53] | 2023 | USA | hiPSC | cell |  |  | Genetic analysis | Genetic data | RNA sequences | SVM, maximum relevance minimum redundancy | ML and Network Analysis revealed panels of gene features differently expressed in healthy hiPSCs and human induced pluripotent stem cell-derived cardiomyocytes (hiPSC-CMs) of different races and sexes | Accuracy over 90% |
| 54 | Wei Feng [54] | 2022 | USA | hiPSC | organoid | cardiomyoctes |  | Genetic analysis | Genetic data | RNA sequences | random forest | Computational phenotyping approach can be used to compare wild type and genetically modified organoids. | Accuracy over 90% |
| 55 | Henry Joutsijoki [55] | 2020 | Europe | hiPSC | cell | cardiomyocytes |  | Cell function monitoring | Cell signal | Ca signal | SVM | ML method both HCMM and HCMT mutations as well as LQT1 and LQT2 disease types could be separated from each other by Ca2þ transient signals with high accuracy | Accuracy is over 80% |
| 56 | Hongbin Yang [56] | 2022 | UK | hiPSC | cell | cardiomyocytes |  | Cell function monitoring | Cell signal | Ca signal | random forest | ML is able to automate the assessment of cardiovascular liability from waveform data reducing any risk of user-to user variability and bias | AUC= 0.86 |
| 57 | Hyun Hwang [57] | 2020 | USA | hiPSC | cell | cardiomyocytes |  | Cell function monitoring | Cell signal | Ca signal | SVM | ML can aid a busy human-expert to eventually fully replicating the decision-making of a humanexpert on all patterns of Ca2+ transient signa | Accuracy was over 80% |
| 58 | Jeremy K.S. Pang [58] | 2022 | Singapore | hiPSC | cell | cardiomyocytes |  | Cell function monitoring | Cell signal | Ca signal | SVM | ML can be used to delectrophysiological data from CM | the multi-class models achieved >90% accuracy in predicting arrhythmia presence and type |
| 59 | Martti Juhola [59] | 2018 | Europe | iPSC | cell | cardiomyocytes |  | Cell function monitoring | Cell signal | Ca signal | SVM, random forest | ML methodology appears to be a powerful means to accurately categorize iPSC-CMs and could provide effective methods for diagnostic purposes in the future | the best classification accuracy obtained was still high: approximately 79% |
| 60 | Nicholas J. Schaub [60] | 2020 | USA | iPSC | cell | retinal |  | Cell function monitoring | image | action potential | CNN | DL can identify shape and texture features of single cells that were used to predict tissue function and iPSC donor identity | Accuracy over 75% |
| 61 | Parya Aghasafari [61] | 2021 | USA | iPSC | cell | cardiomyocytes |  | Cell function monitoring | Cell signal | Ca signal | CNN | DL network can predict the impact of electrophysiological function of iPSC | Accuracy over 90% |
| 62 | Utkarsh Tripathi [62] | 2022 | Japan | hiPSC | cell | neuron |  | Cell function monitoring | Cell signal | MEA | SVM, random forest | AI significantly improving the ability to discriminate between different cell type | Accuracy 75-90% |
| 63 | Adam Witmer [63] | 2018 | USA | hiPSC | cell | neuron | Huntington | Disease modelling | image | Identify disease cell | CNN | Automated detection of cellular behavior changes using pattern recognition and deep learning | ROC AUC of 87 |
| 64 | Keiko Imamura [64] | 2021 | Japan | hiPSC | cell | neuron | Amyotrophic Lateral Sclerosis | Disease modelling | image | Identify disease cell | CNN | DL algorithm with iPSC technology could support the diagnosis and may provide proactive treatment of ALS | AUC was 0.97 |
| 65 | Martti Juhola [65] | 2021 | Europe | iPSC | cell | cardiomyocytes | dilated cardiomyopathy and LQT 2 | Disease modelling | Cell signal | Identify disease cell | SVM, random forest | ML can attain good disease classification | Classification accuracy of above 70 % |
| 66 | Martti Juhola [66] | 2022 | Europe | hiPSC | cell | cardiomyocytes | dilated cardiomyopathy, LQT 1 and 2, Brugada syndrome, ventricular arrhythmia | Disease modelling | Cell signal | Identify disease cell | CNN, random forest | ML can classify the seven diseases and healthy controls with machine learning | Not report |
| 67 | Andrew Kowalczews [67] | 2022 | USA | hiPSC | cell | iPS-CM |  | Drug modelling | Cell signal | Drug testing | CNN, SVM, random forests, t-SNE, | ML algorithms were able to distinguish the utilized drug with 80% accuracy | Treatment determination: over 90%, toxicity prediction: 100%, drug classification: 62.9% - 80% |
| 68 | Anna S. Monzel [68] | 2020 | Europe | hiPSC | organoid | Mid brain organoid | Parkinson | Drug modelling | image | Toxicity prediction | random forest | ML was valuable tool for in vitro modeling and to test putative neurotoxic compounds. | Neurotoxicity accuracy of 85%, prediction of disease state: 93% |
| 69 | Christopher Heylman [69] | 2015 | USA | hiPSC | cell | iPS-CM |  | Drug modelling | Cell signal | Drug testing | decision trees | ML algorithm can accurately assess, classify, and predict hiPS-CM membrane depolarization following exposure to chronotropic drugs | The overall accuracy rate is over 70% |
| 70 | Eugene K. Lee [70] | 2015 | USA | hiPSC | cell | iPS-CM |  | Drug modelling | image | Drug testing | SVM | ML is effectivef for drug screening purposes | Accuracies above 78% |
| 71 | Francis Grafton [71] | 2021 | USA | iPSCs | cell | iPS-CM |  | Drug modelling | Cell signal | Toxicity prediction | CNN | DL is an effective way to interrogate cellular phenotypes and identify drugs that may protect against diseased phenotypes and deleterious mutations | Accuracies above 90% |
| 72 | Hongbin Yang [72] | 2022 | UK | hiPSC | cell | iPS-CM |  | Drug modelling | Cell signal | Drug testing | random forest | ML can be used to screen cardiac risk in early drug discovery | The sensitivity was very high (from 0.90 to 0.94), while the specificity was relatively low (from 0.59 to 0.67) |
| 73 | Karthikeyan Kandasamy [73] | 2015 | Singapore | hiPSC | cell | iPS-renal cell |  | Drug modelling | Biological information | Toxicity prediction | random forest | Injury mechanisms and drug-induced cellular pathways could be reliably identified by using automated cellular imaging | Training accuracy was 99.8% and test accuracy was 87.0% |
| 74 | Manuela Jaklin [74] | 2022 | Europe | hiPSC | embroid | embroid |  | Drug modelling | image | Toxicity prediction | random forest | ML represent a new tool and a valuable resource for drug teratogenicity assessment. | Accuracy of 69% (specificity: 53%, sensitivity: 79%) |
| 75 | Mahnaz Maddah [75] | 2020 | USA | hiPSC | cell | iPS-CM and hepatocyte |  | Drug modelling | image | Toxicity prediction | CNN | DL can capture the subtle morphological changes that are early signs of toxicity in both hepatocytes and cardiomyocytes. | Not report |
| 76 | Martti Juhola [76] | 2021 | Europe | iPSC | cell | iPS-CM |  | Drug modelling | Cell signal | Drug testing | naïve Baye, random forests | ML create a very valuable and efficient platform to individualize medication in addition to drug screening and cardiotoxicity studies | Accuracy of 78.9%, but the imbalanced sensitivities of 90.8% for the merged responders and semi-responders and 53.7% for the non-responders. |
| 77 | N. Matsuda [77] | 2022 | Japan | iPSC | cell | iPS-neuron |  | Drug modelling | image | Drug testing | CNN | AI can identify seizure liability concentration-dependence, rank the level of seizure liability based on the seizure liability probability, and identify the mechanism of the action of compounds | Mean accuracy of 99.9 ± 0.1% |
| 78 | Tadashi Hidaka [78] | 2020 | Japan | hiPSC | cell | iPS-neuron |  | Drug modelling | Biological information | Drug testing | SVM, k-nearest neighbor, random forest | ML predicted hits with new chemotypes among millions of compounds for ALS therapeutics using a panel of large numbers of ALS patient-derived induced pluripotent stem cell models | AUC > 0.7 |
| 79 | Yuki Hanafusa [79] | 2023 | Japan | hiPSC | cell | iPS-neuron |  | Drug modelling | image | Drug testing | random forest | ML can be a powerful tool to reveal the hidden internal alterations of iNSCs by neuronal effective substances | Accuracy over 89% |

**S2 Table.** Quality of included studies

| **No** | **First author** | **Available code and data** | **Clear description of work flow** | **Hyperparameter tuning** | **Data split** | **Model Interpretability** | **Performance measures** | **Best practice report** |
| --- | --- | --- | --- | --- | --- | --- | --- | --- |
| 1 | Aixia Sun [1] | Not report | Yes | Not report | Not report | Not report | Yes | Not report |
| 2 | Anna Skorska [2] | Yes | Not report | Not report | Not report | Not report | No | Not report |
| 3 | Aurore Vuidel [3] | Yes | Yes | Yes | Yes | Yes | Yes | Yes |
| 4 | Bianca Williams [4] | Yes | Yes | Yes | Yes | Yes | Yes | Not report |
| 5 | Bo Jiang [5] | Not report | Yes | Yes | Not report | Not report | Yes | Not report |
| 6 | Brodie Fischbacher [6] | Yes | Yes | Yes | Yes | Yes | Yes | Not report |
| 7 | Cesar A. Patino [7] | Not report | Yes | Not report | Not report | Yes | Yes | Not report |
| 8 | Cesar A. Patino [8] | Not report | Yes | Not report | Not report | Not report | No | Not report |
| 9 | Chia-Chen Hsu [9] | Yes | Yes | Yes | Yes | Yes | Yes | Not report |
| 10 | Chung-Yueh Lien [10] | Not report | Yes | Yes | Yes | Yes | Yes | Not report |
| 11 | Colombine Verzat [11] | Yes | Yes | Yes | Yes | Yes | No | Not report |
| 12 | Dai Kusumoto [12] | Yes | Yes | Yes | Yes | Yes | Yes | Not report |
| 13 | David A. Joy [13] | Yes | Yes | Yes | Yes | Yes | Yes | Not report |
| 14 | Diogo Teles [14] | Not report | Yes | Yes | Yes | Yes | Yes | Not report |
| 15 | Jianying Guo [15] | Yes | Yes | Yes | Yes | Yes | Yes | Not report |
| 16 | Haishan Zhang [16] | Not report | Yes | Yes | Yes | Yes | Yes | Not report |
| 17 | Henry Joutsijoki [17] | Not report | Not report | Yes | Yes | Yes | Yes | Not report |
| 18 | Henry Joutsijoki [18] | Not report | Yes | Yes | Yes | Yes | Yes | Not report |
| 19 | Kaivalya Molugu [19] | Yes | Not report | Yes | Not report | Yes | Yes | Not report |
| 20 | Kazuaki Tokunaga [20] | Not report | Not report | Not report | Not report | Not report | Yes | Not report |
| 21 | Ke Fan [21] | Not report | Yes | Not report | Yes | Yes | Yes | Not report |
| 22 | Ke Ye [22] | Yes | Yes | Yes | Yes | Yes | Yes | Not report |
| 23 | Ken Orita [23] | Not report | Yes | Yes | Yes | Yes | Yes | Not report |
| 24 | Ken Orita [24] | Not report | Yes | Yes | Yes | Yes | Yes | Not report |
| 25 | Keonhyeok Park [25] | Not report | Yes | Yes | Yes | Yes | Yes | Not report |
| 26 | Kimerly A. Powell [26] | Not report | Yes | Yes | Yes | Yes | No | Not report |
| 27 | Letao Yang [27] | Not report | Yes | Yes | Yes | Yes | No | Not report |
| 28 | Mahnaz Maddah [28] | Not report | Yes | Not report | Yes | Not report | Yes | Not report |
| 29 | Minjae Kim [29] | Yes | Yes | Yes | Yes | Yes | Yes | Not report |
| 30 | Muthu Subash Kavitha [30] | Not report | Yes | Yes | Yes | Yes | Yes | Not report |
| 31 | Na Ta [31] | Not report | Yes | Yes | Yes | Yes | Yes | Not report |
| 32 | Prithvijit Mukherjee [32] | Not report | Yes | Yes | Yes | Yes | Yes | Not report |
| 33 | Quinton Smith [33] | Not report | Yes | Not report | Not report | Not report | Yes | Not report |
| 34 | Sally Esmail [34] | Not report | Yes | Not report | Not report | Not report | Yes | Not report |
| 35 | Sally Esmail [35] | Not report | Yes | Not report | Not report | Not report | Yes | Not report |
| 36 | Saori Aida [36] | Not report | Yes | Yes | Not report | Yes | No | Not report |
| 37 | Scott Atwell [37] | Yes | Yes | Yes | Yes | Yes | Yes | Not report |
| 38 | Slo-Li Chu [38] | Not report | Yes | Yes | Yes | Yes | Yes | Not report |
| 39 | Takashi Waku [39] | Not report | Not report | Yes | Not report | Not report | Yes | Not report |
| 40 | Tobias Piotrowski [40] | Not report | Yes | Yes | Yes | Yes | Yes | Not report |
| 41 | Toshiro Iwagawa [41] | Not report | Not report | Yes | Not report | Not report | Yes | Not report |
| 42 | Yohei Hayashi [42] | Yes | Yes | Yes | Yes | Not report | No | Not report |
| 43 | Yuan-Hsiang Chang [43] | Not report | Yes | Yes | Yes | Not report | Yes | Not report |
| 44 | Wayne R. Danter [44] | Not report | Yes | Not report | Not report | Not report | Yes | Not report |
| 45 | Alberto Catanese [45] | Not report | Not report | Not report | Not report | Not report | No | Not report |
| 46 | Asato Sekiya [46] | Not report | Not report | Not report | Not report | Not report | No | Not report |
| 47 | Boqiao Lai [47] | Not report | Yes | Yes | Not report | Yes | Yes | Not report |
| 48 | C Bardy [48] | Not report | Yes | Yes | Yes | Not report | Yes | Not report |
| 49 | Christina V. Theodoris [49] | Not report | Not report | Not report | Not report | Not report | Yes | Not report |
| 50 | Koichiro Nishino [50] | Not report | Yes | Yes | Yes | Yes | Yes | Not report |
| 51 | Quan H. Nguyen [51] | Yes | Yes | Yes | Yes | Yes | Yes | Not report |
| 52 | S John Liu [52] | Not report | Not report | Not report | Not report | Not report | Yes | Not report |
| 53 | Thong Ba Nguyen [53] | Not report | Yes | Not report | Not report | Not report | Yes | Not report |
| 54 | Wei Feng [54] | Yes | Yes | Yes | Yes | Yes | Yes | Not report |
| 55 | Henry Joutsijoki [55] | Not report | Yes | Yes | Not report | Not report | Yes | Not report |
| 56 | Hongbin Yang [56] | Yes | Yes | Yes | Yes | Not report | Yes | Not report |
| 57 | Hyun Hwang [57] | Yes | Not report | Yes | Yes | Not report | Yes | Not report |
| 58 | Jeremy K.S. Pang [58] | Yes | Yes | Yes | Yes | Yes | Yes | Not report |
| 59 | Martti Juhola [59] | Not report | Yes | Not report | Yes | Not report | Yes | Not report |
| 60 | Nicholas J. Schaub [60] | Not report | Yes | Yes | Yes | Yes | Yes | Not report |
| 61 | Parya Aghasafari [61] | Yes | Yes | Yes | Yes | Yes | Yes | Not report |
| 62 | Utkarsh Tripathi [62] | Not report | Yes | Not report | Not report | Yes | Yes | Not report |
| 63 | Adam Witmer [63] | Not report | Yes | Yes | Yes | Yes | Yes | Not report |
| 64 | Keiko Imamura [64] | Not report | Yes | Yes | Yes | Yes | Yes | Not report |
| 65 | Martti Juhola [65] | Not report | Yes | Yes | Yes | Yes | Yes | Not report |
| 66 | Martti Juhola [66] | Not report | Yes | Yes | Yes | Yes | No | Not report |
| 67 | Andrew Kowalczews [67] | Not report | Yes | Yes | Yes | Yes | Yes | Not report |
| 68 | Anna S. Monzel [68] | Yes | Not report | Yes | Not report | Not report | Yes | Not report |
| 69 | Christopher Heylman [69] | Yes | Yes | Yes | Yes | Yes | Yes | Not report |
| 70 | Eugene K. Lee [70] | Not report | Yes | Yes |  |  | Yes | Not report |
| 71 | Francis Grafton [71] | Not report | Yes | Yes | Yes | Yes | Yes | Yes |
| 72 | Hongbin Yang [72] | Not report | Yes | Yes | Yes | Yes | Yes | Not report |
| 73 | Karthikeyan Kandasamy [73] | Not report | Yes | Yes | Yes | Not report | Yes | Not report |
| 74 | Manuela Jaklin [74] | Not report | Yes | Yes | Yes | Yes | Yes | Not report |
| 75 | Mahnaz Maddah [75] | Not report | Yes | Yes | Yes | Yes | Yes | Not report |
| 76 | Martti Juhola [76] | Not report | Yes | Yes | Yes | Yes | Yes | Not report |
| 77 | N. Matsuda [77] | Not report | Yes | Yes | Not report | Not report | Yes | Not report |
| 78 | Tadashi Hidaka [78] | Yes | Yes | Yes | Yes | Yes | Yes | Not report |
| 79 | Yuki Hanafusa [79] | Not report | Yes | Yes |  | Yes | Yes | Not report |

**List of included studies**

1. Sun, A., et al., *3D in vivo Magnetic Particle Imaging of Human Stem Cell-Derived Islet Organoid Transplantation Using a Machine Learning Algorithm.* Front Cell Dev Biol, 2021. **9**: p. 704483.

2. Skorska, A., et al., *Monitoring the maturation of the sarcomere network: a super-resolution microscopy-based approach.* Cell Mol Life Sci, 2022. **79**(3): p. 149.

3. Vuidel, A., et al., *High-content phenotyping of Parkinson's disease patient stem cell-derived midbrain dopaminergic neurons using machine learning classification.* Stem Cell Reports, 2022. **17**(10): p. 2349-2364.

4. Williams, B., et al., *Prediction of Human Induced Pluripotent Stem Cell Cardiac Differentiation Outcome by Multifactorial Process Modeling.* Front Bioeng Biotechnol, 2020. **8**: p. 851.

5. Jiang, B., et al. *Application of Support Vector Machine to Recognize Trans-differentiated Neural Progenitor Cells for Bright-Field Microscopy*. in *2015 Fifth International Conference on Instrumentation and Measurement, Computer, Communication and Control (IMCCC)*. 2015.

6. Fischbacher, B., et al., *Modular deep learning enables automated identification of monoclonal cell lines.* bioRxiv, 2020: p. 2020.12.28.424610.

7. Patino, C.A., et al., *Deep Learning and Computer Vision Strategies for Automated Gene Editing with a Single-Cell Electroporation Platform.* SLAS Technol, 2021. **26**(1): p. 26-36.

8. Patino, C.A., et al., *Multiplexed high-throughput localized electroporation workflow with deep learning-based analysis for cell engineering.* Sci Adv, 2022. **8**(29): p. eabn7637.

9. Hsu, C.C., et al., *A single-cell Raman-based platform to identify developmental stages of human pluripotent stem cell-derived neurons.* Proc Natl Acad Sci U S A, 2020. **117**(31): p. 18412-18423.

10. Lien, C.Y., et al., *Recognizing the Differentiation Degree of Human Induced Pluripotent Stem Cell-Derived Retinal Pigment Epithelium Cells Using Machine Learning and Deep Learning-Based Approaches.* Cells, 2023. **12**(2).

11. Verzat, C., et al., *Image-based deep learning reveals the responses of human motor neurons to stress and VCP-related ALS.* Neuropathol Appl Neurobiol, 2022. **48**(2): p. e12770.

12. Kusumoto, D., et al., *Automated Deep Learning-Based System to Identify Endothelial Cells Derived from Induced Pluripotent Stem Cells.* Stem Cell Reports, 2018. **10**(6): p. 1687-1695.

13. Joy, D.A., A.R.G. Libby, and T.C. McDevitt, *Deep neural net tracking of human pluripotent stem cells reveals intrinsic behaviors directing morphogenesis.* Stem Cell Reports, 2021. **16**(5): p. 1317-1330.

14. Teles, D., et al., *Machine Learning Techniques to Classify Healthy and Diseased Cardiomyocytes by Contractility Profile.* ACS Biomater Sci Eng, 2021. **7**(7): p. 3043-3052.

15. Guo, J., et al., *Machine learning-assisted high-content analysis of pluripotent stem cell-derived embryos in vitro.* Stem Cell Reports, 2021. **16**(5): p. 1331-1346.

16. Zhang, H., et al., *A novel machine learning based approach for iPS progenitor cell identification.* PLoS Comput Biol, 2019. **15**(12): p. e1007351.

17. Joutsijoki, H., et al. *Histogram-based classification of iPSC colony images using machine learning methods*. in *2014 IEEE International Conference on Systems, Man, and Cybernetics (SMC)*. 2014.

18. Joutsijoki, H., et al., *Machine Learning Approach to Automated Quality Identification of Human Induced Pluripotent Stem Cell Colony Images.* Comput Math Methods Med, 2016. **2016**: p. 3091039.

19. Molugu, K., et al., *Label-Free Imaging to Track Reprogramming of Human Somatic Cells.* GEN Biotechnol, 2022. **1**(2): p. 176-191.

20. Tokunaga, K., et al., *Computational image analysis of colony and nuclear morphology to evaluate human induced pluripotent stem cells.* Sci Rep, 2014. **4**: p. 6996.

21. Fan, K., et al., *A Machine Learning Assisted, Label-free, Non-invasive Approach for Somatic Reprogramming in Induced Pluripotent Stem Cell Colony Formation Detection and Prediction.* Scientific Reports, 2017. **7**(1): p. 13496.

22. Ye, K., et al., *Reproducible production and image-based quality evaluation of retinal pigment epithelium sheets from human induced pluripotent stem cells.* Scientific Reports, 2020. **10**(1): p. 14387.

23. Orita, K., et al., *Machine-learning-based quality control of contractility of cultured human-induced pluripotent stem-cell-derived cardiomyocytes.* Biochem Biophys Res Commun, 2020. **526**(3): p. 751-755.

24. Orita, K., et al., *Deep learning-based quality control of cultured human-induced pluripotent stem cell-derived cardiomyocytes.* J Pharmacol Sci, 2019. **140**(4): p. 313-316.

25. Park, K., et al., *Deep learning predicts the differentiation of kidney organoids derived from human induced pluripotent stem cells.* Kidney Res Clin Pract, 2023. **42**(1): p. 75-85.

26. Powell, K.A., et al., *Automated human induced pluripotent stem cell colony segmentation for use in cell culture automation applications.* SLAS Technol, 2023. **28**(6): p. 416-422.

27. Yang, L., et al., *High-Content Screening and Analysis of Stem Cell-Derived Neural Interfaces Using a Combinatorial Nanotechnology and Machine Learning Approach.* Research (Wash D C), 2022. **2022**: p. 9784273.

28. Maddah, M. and K. Loewke, *Automated, non-invasive characterization of stem cell-derived cardiomyocytes from phase-contrast microscopy.* Med Image Comput Comput Assist Interv, 2014. **17**(Pt 1): p. 57-64.

29. Kim, M., et al., *Prediction of Stem Cell State Using Cell Image-Based Deep Learning.* Advanced Intelligent Systems, 2023. **5**(7): p. 2300017.

30. Kavitha, M.S., T. Kurita, and B.C. Ahn, *Critical texture pattern feature assessment for characterizing colonies of induced pluripotent stem cells through machine learning techniques.* Comput Biol Med, 2018. **94**: p. 55-64.

31. Ta, N., et al., *Mining Key Regulators of Cell Reprogramming and Prediction Research Based on Deep Learning Neural Networks.* IEEE Access, 2020. **8**: p. 23179-23185.

32. Mukherjee, P., et al., *Deep Learning-Assisted Automated Single Cell Electroporation Platform for Effective Genetic Manipulation of Hard-to-Transfect Cells.* Small, 2022. **18**(20): p. e2107795.

33. Smith, Q., et al., *Cytoskeletal tension regulates mesodermal spatial organization and subsequent vascular fate.* Proceedings of the National Academy of Sciences, 2018. **115**(32): p. 8167-8172.

34. Esmail, S. and W.R. Danter, *DeepNEU: Artificially Induced Stem Cell (aiPSC) and Differentiated Skeletal Muscle Cell (aiSkMC) Simulations of Infantile Onset POMPE Disease (IOPD) for Potential Biomarker Identification and Drug Discovery.* Front Cell Dev Biol, 2019. **7**: p. 325.

35. Esmail, S. and W. Danter, *Viral pandemic preparedness: A pluripotent stem cell-based machine-learning platform for simulating SARS-CoV-2 infection to enable drug discovery and repurposing.* Stem Cells Transl Med, 2021. **10**(2): p. 239-250.

36. Aida, S., et al., *Conditional Generative Adversarial Networks to Model iPSC-Derived Cancer Stem Cells.* Journal of Advanced Computational Intelligence and Intelligent Informatics, 2020. **24**(1): p. 134-141.

37. Atwell, S., et al., *Label-free imaging of 3D pluripotent stem cell differentiation dynamics on chip.* Cell Rep Methods, 2023. **3**(7): p. 100523.

38. Chu, S.L., et al., *Human induced pluripotent stem cell formation and morphology prediction during reprogramming with time-lapse bright-field microscopy images using deep learning methods.* Comput Methods Programs Biomed, 2023. **229**: p. 107264.

39. Wakui, T., et al., *Method for evaluation of human induced pluripotent stem cell quality using image analysis based on the biological morphology of cells.* J Med Imaging (Bellingham), 2017. **4**(4): p. 044003.

40. Piotrowski, T., et al., *Deep-learning-based multi-class segmentation for automated, non-invasive routine assessment of human pluripotent stem cell culture status.* Comput Biol Med, 2021. **129**: p. 104172.

41. Iwagawa, T., et al., *Evaluation of CRISPR/Cas9 exon-skipping vector for choroideremia using human induced pluripotent stem cell-derived RPE.* J Gene Med, 2023. **25**(2): p. e3464.

42. Hayashi, Y., et al., *Automated adherent cell elimination by a high-speed laser mediated by a light-responsive polymer.* Communications Biology, 2018. **1**(1): p. 218.

43. Yuan-Hsiang, C., et al., *Human induced pluripotent stem cell region recognition in microscopy images using Convolutional Neural Networks.* Annu Int Conf IEEE Eng Med Biol Soc, 2017. **2017**: p. 4058-4061.

44. Danter, W.R., *DeepNEU: cellular reprogramming comes of age - a machine learning platform with application to rare diseases research.* Orphanet J Rare Dis, 2019. **14**(1): p. 13.

45. Catanese, A., et al., *Multiomics and machine-learning identify novel transcriptional and mutational signatures in amyotrophic lateral sclerosis.* Brain, 2023. **146**(9): p. 3770-3782.

46. Sekiya, A., et al., *Variation of DNA methylation on the IRX1/2 genes is responsible for the neural differentiation propensity in human induced pluripotent stem cells.* Regen Ther, 2022. **21**: p. 620-630.

47. Lai, B., et al., *Annotating functional effects of non-coding variants in neuropsychiatric cell types by deep transfer learning.* PLoS Comput Biol, 2022. **18**(5): p. e1010011.

48. Bardy, C., et al., *Predicting the functional states of human iPSC-derived neurons with single-cell RNA-seq and electrophysiology.* Mol Psychiatry, 2016. **21**(11): p. 1573-1588.

49. Theodoris, C.V., et al., *Network-based screen in iPSC-derived cells reveals therapeutic candidate for heart valve disease.* Science, 2021. **371**(6530).

50. Nishino, K., et al., *Identification of an epigenetic signature in human induced pluripotent stem cells using a linear machine learning model.* Hum Cell, 2021. **34**(1): p. 99-110.

51. Nguyen, Q.H., et al., *Single-cell RNA-seq of human induced pluripotent stem cells reveals cellular heterogeneity and cell state transitions between subpopulations.* Genome Res, 2018. **28**(7): p. 1053-1066.

52. Liu, S.J., et al., *CRISPRi-based genome-scale identification of functional long noncoding RNA loci in human cells.* Science, 2017. **355**(6320).

53. Nguyen, T.B., et al., *Harshening stem cell research and precision medicine: The states of human pluripotent cells stem cell repository diversity, and racial and sex differences in transcriptomes.* Front Cell Dev Biol, 2022. **10**: p. 1071243.

54. Feng, W., et al., *Computational profiling of hiPSC-derived heart organoids reveals chamber defects associated with NKX2-5 deficiency.* Communications Biology, 2022. **5**(1): p. 399.

55. Joutsijoki, H., et al., *Separation of HCM and LQT Cardiac Diseases with Machine Learning of Ca2+ Transient Profiles.* Methods Inf Med, 2019. **58**(4-05): p. 167-178.

56. Yang, H., et al., *Deriving waveform parameters from calcium transients in human iPSC-derived cardiomyocytes to predict cardiac activity with machine learning.* Stem Cell Reports, 2022. **17**(3): p. 556-568.

57. Hwang, H., et al., *Machine learning identifies abnormal Ca(2+) transients in human induced pluripotent stem cell-derived cardiomyocytes.* Sci Rep, 2020. **10**(1): p. 16977.

58. Pang, J.K.S., et al., *Characterizing arrhythmia using machine learning analysis of Ca(2+) cycling in human cardiomyocytes.* Stem Cell Reports, 2022. **17**(8): p. 1810-1823.

59. Juhola, M., et al., *Detection of genetic cardiac diseases by Ca2+ transient profiles using machine learning methods.* Scientific Reports, 2018. **8**(1): p. 9355.

60. Schaub, N.J., et al., *Deep learning predicts function of live retinal pigment epithelium from quantitative microscopy.* J Clin Invest, 2020. **130**(2): p. 1010-1023.

61. Aghasafari, P., et al., *A deep learning algorithm to translate and classify cardiac electrophysiology.* Elife, 2021. **10**.

62. Tripathi, U., et al., *Information theory characteristics improve the prediction of lithium response in bipolar disorder patients using a support vector machine classifier.* Bipolar Disord, 2023. **25**(2): p. 110-127.

63. Witmer, A. and B. Bhanu, *Multi-label Classification of Stem Cell Microscopy Images Using Deep Learning*. 2018. 1408-1413.

64. Imamura, K., et al., *Prediction Model of Amyotrophic Lateral Sclerosis by Deep Learning with Patient Induced Pluripotent Stem Cells.* Ann Neurol, 2021. **89**(6): p. 1226-1233.

65. Juhola, M., et al., *On computational classification of genetic cardiac diseases applying iPSC cardiomyocytes.* Comput Methods Programs Biomed, 2021. **210**: p. 106367.

66. Juhola, M., et al., *A method to measure data complexity of a complicated medical data set.* International Journal of Imaging Systems and Technology, 2022. **32**(6): p. 1822-1831.

67. Kowalczewski, A., et al., *Integrating nonlinear analysis and machine learning for human induced pluripotent stem cell-based drug cardiotoxicity testing.* J Tissue Eng Regen Med, 2022. **16**(8): p. 732-743.

68. Monzel, A.S., et al., *Machine learning-assisted neurotoxicity prediction in human midbrain organoids.* Parkinsonism Relat Disord, 2020. **75**: p. 105-109.

69. Heylman, C., et al., *Supervised Machine Learning for Classification of the Electrophysiological Effects of Chronotropic Drugs on Human Induced Pluripotent Stem Cell-Derived Cardiomyocytes.* PLoS One, 2015. **10**(12): p. e0144572.

70. Lee, E.K., et al., *Machine learning plus optical flow: a simple and sensitive method to detect cardioactive drugs.* Sci Rep, 2015. **5**: p. 11817.

71. Grafton, F., et al., *Deep learning detects cardiotoxicity in a high-content screen with induced pluripotent stem cell-derived cardiomyocytes.* Elife, 2021. **10**.

72. Yang, H., et al., *Prediction of inotropic effect based on calcium transients in human iPSC-derived cardiomyocytes and machine learning.* Toxicol Appl Pharmacol, 2023. **459**: p. 116342.

73. Kandasamy, K., et al., *Prediction of drug-induced nephrotoxicity and injury mechanisms with human induced pluripotent stem cell-derived cells and machine learning methods.* Sci Rep, 2015. **5**: p. 12337.

74. Jaklin, M., et al., *Optimization of the TeraTox Assay for Preclinical Teratogenicity Assessment.* Toxicol Sci, 2022. **188**(1): p. 17-33.

75. Maddah, M., et al., *Quantifying drug-induced structural toxicity in hepatocytes and cardiomyocytes derived from hiPSCs using a deep learning method.* J Pharmacol Toxicol Methods, 2020. **105**: p. 106895.

76. Juhola, M., et al., *Analysis of Drug Effects on iPSC Cardiomyocytes with Machine Learning.* Ann Biomed Eng, 2021. **49**(1): p. 129-138.

77. Matsuda, N., et al., *Raster plots machine learning to predict the seizure liability of drugs and to identify drugs.* Sci Rep, 2022. **12**(1): p. 2281.

78. Hidaka, T., et al., *Prediction of Compound Bioactivities Using Heat-Diffusion Equation.* Patterns (N Y), 2020. **1**(9): p. 100140.

79. Hanafusa, Y., A. Shiraishi, and F. Hattori, *Machine learning discriminates P2X7-mediated intracellular calcium sparks in human-induced pluripotent stem cell-derived neural stem cells.* Scientific Reports, 2023. **13**(1): p. 12673.
