# Supplementary material for: The use of artificial intelligence in induced pluripotent stem cell-based technology over 10-year period: A systematic scoping review
Source: PLoS One. 2024 May 21;19(5):e0302537. doi: 10.1371/journal.pone.0302537 (PMC11108174; doi:10.1371/journal.pone.0302537)
Supplement: Attachment — Submitted filename: PLoS One review.docx [file pone.0302537.s003.docx]

This manuscript by Vo *et al*. provides a comprehensive review of the application of artificial intelligence (AI) technologies, particularly machine learning and deep learning, in the processing of induced pluripotent stem cells (iPSCs). The authors have systematically reviewed 79 studies, highlighting the increasing trend of research in this area, with the United States being a leading contributor. The paper underscores the diverse applications of AI in iPSC technology, including cell type classification, disease-specific phenotype assessment in iPSC-derived cells, and facilitation of drug screening. The authors conclude by emphasizing the potential of AI technologies in advancing regenerative and personalized medicine, despite being in its early stages. They also highlight the challenges and opportunities in this field, suggesting that AI holds significant promise for enhancing our understanding of disease progression and development.

Although scientists have already written similar reviews on the interface of these two technologies in the last years (i.e. <http://dx.doi.org/10.2174/1389201021666201007122524> or <https://doi.org/10.3390%2Fbiomimetics8050442>), this is a fast paced field and frequent literature reviews can help to understand progress. In this case the systematic nature of the review is also intriguing.

Before this work can be published, several aspects should be addressed (see major comments). Although it has a good structure, in general, the manuscript lacks sufficient depth for a scientific literature review. Key studies are rarely described in detail and several claims especially around human health and drug discovery improvements are made without clear connection to the literature.

Major comments:

1. Since the scope of the manuscript is the use of different AI-related technologies in the iPSC-field, I would expect a detailed and original figure giving a graphical summary of the frequency of the most commonly applied ML and DL algorithms. Which algorithms are used mostly? Is there a preference for certain algorithms in certain cell types? What are the main roles of the used algorithms (feature extraction, classification, regression, etc.)? The authors provide an excellent supplementary Table S1, which is a bit hidden, but is the core piece of this work. These results need to be more graphically visible. I would like to make the following proposition: First, merge Figure 2 and 3 into a new Figure 2 since these figures describe less interesting metadata. Second, use the data in Table S1 to create a new and insightful Figure 3 as described above.
2. Figures 4 and 5 have just been copied from two other publications and individually offer little to no new information. Also in this case I would encourage the authors to create a new Figure 4 by themselves which merges the information of the two current figures. In the new figure highlight specifically (and based on the literature) which kind if iPSC derived data (image features, molecular data, functional data) is generated or processed by which specific ML/DL algorithms and for what purpose (mentioned throughout the review). This will give a reader a clear understanding of the current literature. The current figures are just too general.
3. The authors make several strong claims about the use of AI and stem cell technology to improve human health. Although I agree that fundamental research has profited a lot, key clinical breakthroughs enabled by AI have yet to emerge (a new approved drug, stem cell therapy, approved disease models). For example, applying an AI algorithm to extract phenotypes from iPSC-cardiomyocytes is not yet a breakthrough for patients. Please back up these claims with literature or remove them. In general, go more in detail into key studies.

For example:

- “Recent advancement in artificial intelligence (AI) technologies, including machine learning, deep learning, and convolutional neural networks (CNN), has become fundamental in enhancing iPSC therapy.”

What is iPSC therapy (do you mean cell replacement?)? How has field profited exactly? Please go into key studies.

- “Recently, the application of Artificial Intelligence (AI) has significantly expanded, finding usage not just in regenerative medicine but across various domains in the medical field.”

Please be specific and go in detail into studies that describe this.

- “These findings highlighted that network-based screening using artificial intelligence technologies is a powerful method for identifying molecules that address disrupted gene networks in human diseases, offering promising therapy for in vivo validation.”

Therapy for in vivo validation does not make sense. It should be candidate molecules for in vivo validation.

- “These studies highlight the considerable potential of AI technology in the classification and assessment of iPSC-derived cells, significantly contributing to the advancement of disease modelling and drug development processes.”

How are drug development processes exactly positively impacted? Please be very specific and go more into individual studies.

- “This underscores AI's capability to analyse disease phenotypes, further emphasizing its role in advancing medical research and drug discovery.”

How exactly has this aided drug discovery? Which molecules in clinical phases are coming from an AI-aided iPSC system? Please be specific and mention the related work.

- “These developments highlight the significant impact of AI in drug development and disease research, emphasizing its capacity to provide innovative and efficient solutions in medical fields.”
- “The advancement of AI-driven systems such as Deep-SeSMo and SSGraphCPI demonstrates their potential in identifying new therapeutic agents to treat various diseases.”

That might be true, but please give detailed examples from the actual studies.

- “[…] has become fundamental in enhancing iPSC therapy.”

4. The above mentioned sections should be removed, toned down or ideally backed up with detailed literature information. The preferred option would be to add a new dedicated section on how iPSC-derived and ML/DL processed data has contributed to the drug development process. This would be in line with the existing sections on Cell culture, Disease models, Genetic analysis, Functional monitoring etc.
5. A very big issue in the field of ML/DL in biology is reproducibility. In would be important to add another figure or table describing which percentage of papers make code and data available and how well the ML methods and workflows are described. Do papers describe whether hyperparameter tuning was performed? How many papers follow best practice guidelines? For example the DOME (data, optimization, model, and evaluation) recommendations (<https://doi.org/10.1038/s41592-021-01205-4>) or similar best practices.

Minor comments:

1. Remove judgemental adjectives such as “impressive” or “notable success”.
2. The Discussion part is in the middle of the manuscript and should be moved more to the end.
3. Below Figure 4 (I believe) the section header “Quality control” is missing. At least I have the impression that this section is on quality control and has no header.
4. In the section “Cell function monitoring” mostly Calcium assays are described. However, microelectrode arrays (MEAs) are also routinely used to measure muscle or neuron electrophysiological activity. This kind of data is also often analyzed by ML. Please include these studies as well. For example: <https://doi.org/10.1038/s41598-022-05697-8> or <https://doi.org/10.3389%2Ffnins.2021.647877>.
5. In the Discussion or Conclusion section go into the fact that ML/DL algorithms (like any technique) are sensitive to artifacts that can sometimes be avoided or at least be controlled for. For example: Plate position (edge effects), clonal effects, biological versus technical replicates (training and testing data from the same replicate?), need for good benchmarking and ground truth data sets.
