# Supplementary material for: The use of artificial intelligence in induced pluripotent stem cell-based technology over 10-year period: A systematic scoping review
Source: PLoS One. 2024 May 21;19(5):e0302537. doi: 10.1371/journal.pone.0302537 (PMC11108174; doi:10.1371/journal.pone.0302537)
Supplement: Attachment — Submitted filename: Response to Reviewers.docx [file pone.0302537.s005.docx]

Thank you for your comprehensive feedback. Here are our responses to the reviewers’ comments.

**Reviewer 1:**

- I would like to propose a change of the main title: The word “utilization” is rarely used. A simple synonym such as “use” would be better.

- In the sub-section header “Main AI algorithm employed in iPSC research” the word “algorithm” should be in its plural form “algorithms”.

- The sub-section header “Applications of AI in iPSC” should be “Applications of AI in iPSC research” or something similar.

- “Fig 1. The application of AI technology in iPSC field” should be “Fig 1. The application of AI technology in the iPSC field.” (Punctuation and the word “the” was missing.

- The authors often use “iPSC” as a synonym for the word “technology”, but iPSC essentially just means “cells”. For example: In the sentence “AI has been instrumental in advancing iPSC development […]” the word “technology” should be added: “AI has been instrumental in advancing iPSC technology development […]”. Please make sure to check this throughout the manuscript.

**Response**: We have changed our manuscript following the reviewer’s suggestion and carefully check the grammar mistakes in our manuscript.
